# Effects of obesity on the lipid and metabolite profiles of young adults by serum ^1^H-NMR spectroscopy

**DOI:** 10.7717/peerj.7137

**Published:** 2019-06-20

**Authors:** Duanghathai Pasanta, Sirirat Chancharunee, Montree Tungjai, Hong Joo Kim, Suchart Kothan

**Affiliations:** 1 Department of Radiologic Technology, Faculty of Associated Medical Sciences, Chiang Mai University, Chiang Mai, Thailand; 2 Department of Chemistry, Faculty of Science, Chiang Mai University, Chiang Mai, Thailand; 3 Department of Physics, Kyungpook National University, Daegu, South Korea

**Keywords:** Young adults, Obesity, Lipids, Overweight, Nuclear magnetic resonance spectroscopy, Metabolite profile, CH_2_ lipids, CH_3_ lipids, HbA1c, PLS-DA

## Abstract

**Background:**

Overweight (OW) is considered a risk for various metabolic diseases. However, its effects as a mechanism that alters the metabolite profiles remain unclear. The purpose of this study is to investigate the effects that OW has on the lipid and metabolite profiles in young adults.

**Methods:**

The serum metabolite profiles of 46 young adults of normal weight and those considered OW were studied by Proton nuclear magnetic resonance spectroscopy (^1^H NMR) technique.

**Results:**

^1^H NMR metabolite analysis shows the alteration of metabolic levels and increased levels of CH_2_ lipids and CH_3_ lipids, which are used as unique biomarkers to identify OW subjects from the normal weight groups.

**Conclusion:**

This present study reveals that OW contributes to the systemic metabolism and the metabolite alteration among young adults. The alteration in serum lipids level could shed the light on metabolic syndrome pathogenesis in young adults and needs further elucidation.

## Introduction

Obesity is one of the world’s leading health problems (Abdou, Magour & Mahmoud, 2009; Dietz, 2017; Hruby & Hu, 2015; Pasanta et al., 2018; Ramachandran & Snehalatha, 2010), and it reduces the quality of life and health for people affected by it. Obesity has been directly associated with 3.4 million deaths each year and is associated with a higher risk of non-communicable diseases such as cardiovascular disease, diabetes, and certain kinds of cancer (Anstee, Targher & Day, 2013; Berger, 2018; Hu et al., 2001; Lim et al., 2012). While existing research is extensive regarding overweight (OW) and its health risk in both children and adults, there are few reports about the transitional age gaps between these two age groups.

Young adulthood is a transitioning period that takes place between adolescence and adulthood. Increasingly, evidence suggests that young adults have higher risks for developing unhealthy lifestyles, may excessively consume food and sugary drinks, and can also make risky decisions regarding their own lives (Aucott et al., 2014). The social, biological, and psychological effects during this phase of life affects the individual well into adulthood (Aucott et al., 2014; Poobalan et al., 2014). This age group reported a higher risk for gaining weight at one kg each year (Poobalan & Aucott, 2016), with a twofold prevalence of OW occurring during this period when compared to younger ages (Dietz, 2017). Young adults between the age of 18–25-years old, or emerging adults, have been shown to have the highest prevalence for OW when compared to other age groups (Mokdad et al., 2003). Moreover, significant weight gain among young adults is associated with a steady increase in weight throughout the later years (Peltzer et al., 2014; Vadeboncoeur, Foster & Townsend, 2016; Vadeboncoeur, Townsend & Foster, 2015). However, the research concerning the young adults aged between 18–25 years old is limited because the nature of this age group is that they are hard to reach when compared to middle age adults and children (Huang et al., 2003; Poobalan et al., 2014).

Metabolomics has proven to be a powerful tool for accurate biochemical mechanism study in humans. The alteration of metabolite levels reflects changes in cellular activity, metabolism, and health status (Gowda & Raftery, 2015). Therefore, a comprehensive metabolite study may help better understand the effects of OW on mechanisms of development and the progression of diseases, leading to a new strategy for screening and prevention. Nuclear magnetic resonance spectroscopy (NMR) is one commonly used technique for human metabolomics study (Gao et al., 2009). NMR is able to give information about many metabolites in serum in one exam without requiring split biochemical tests. Previous studies have shown how weight effects healthy young adults at the age of 16, and at the average age of 32 years old in terms of how weight alters the extensive metabolite levels and how it alters metabolic system (Würtz et al., 2014). However, data from young adults age between 18–25 years old is still lacking, especially considering that the behavior changes that occur mostly develop from their increasingly independent lifestyles during these years (Aucott et al., 2014; World Health Organization, 2000). The purpose of this study is to investigate the lipid and metabolite profiles in serum by ^1^H NMR in young adults ages 18–25 between normal and OW.

## Materials and Methods

The study subjects were recruited via advertisement at clinical service centers located at Chiang Mai province. All procedures were approved by the Ethics Committee of the Faculty of Associated Medical Sciences, Chiang Mai University, Chiang Mai, Thailand (AMSEC-61EX-016). Subjects were recruited with written informed consent obtained after fully understanding the nature of the study. The control group included subjects with normal ranges of body mass index (BMI) between 18.5 and 24.9 kg/m^2^ based on standards set by the World Health Organization (WHO) (World Health Organization, 1995). OW groups included subjects with BMIs in OW ranges (BMI 25.0–29.9 kg/m^2^). Screening tests were first taken for medical history, basic data, and blood laboratory test results to determine inclusion or exclusion for this study. The inclusion criteria are as follows: (1) 19–25 years of age (2) no history of chronic diseases such as diabetes or cardiovascular disease. Subjects were excluded from the study based on the following criteria: taking a drug that may affect lipid levels, blood sugar, or metabolic levels.

The anthropometry of subjects was measured by a single examiner. Height and weight were measured using the same set of equipment for every subject. BMI was calculated by divided body weight in kilograms by height in square meters.

Venous Blood samples were collected after 8–12 h overnight fasting. Blood collection of subjects was done by The Associated Medical Science Clinical Service Center, Chiang Mai University. Intravenous blood of 10 mL was drawn from antecubital veins for biochemical analysis by automated analyzer (ARCHITECT ci8200, Chicago, IL, USA). The blood samples were tested for total cholesterol, high density lipoproteins (HDLs), very low-density lipoproteins (VLDLs), triglycerides, blood glucose, and glycated hemoglobin (HbA1c). Low density lipoprotein (LDL) concentration was calculated from a novel adjustable ratio LDL estimation equation (Friedewald, Levy & Fredrickson, 1972; Martin et al., 2013). Dyslipidemia was determined according to guidelines set by the national cholesterol education project adult treatment panel III with cholesterol ≥ 200 mg/dL, triglyceride ≥ 150 mg/dL, LDL ≥ 130 mg/dL, and HDL ≤ 40 mg/dL (Expert Panel on Detection, Evaluation, and Treatment of High Blood Cholesterol in Adults, 2001). Normal blood glucose level should be between 70 and 100 mg/dL, pre-diabetes is defined by 100–125 mg/dL. Normal HbA1c levels being lower than 6% (American Diabetes Association, 2008).

Blood samples for NMR study were collected from subjects and were centrifuged at 3,500×*g* for 10 min to separate blood serum. The 10 mL of serum samples were lyophilized before NMR study and were then stored at −20 °C until the time of study. Lyophilized serum powder was dissolved with deuterium oxide (Sigma-Aldrich, St. Louis, MO, USA) 500 μL, and then was mixed gently. After that, the homogeneous solution was transferred into a five mm high-quality NMR tube. NMR signals of serum metabolites were collected at 300 K. All samples were performed on a Bruker AVANCE 400 MHz (Bruker Biospin, Rheinstetten, Germany) with 1D water-suppression pulses using pre-saturation pulse sequence. 90°-pulse was applied. The number of signal averages = 16 Spectra in zero to six ppm range were analyzed by TopSpin version 4.0.1 software. The base line and phase were manually corrected and referenced to internal lactate peaks at 4.12 ppm. Metabolites were identified by comparing to existing literature and the human metabolome database (http://www.hmdb.ca) (Du et al., 2014; McKnight et al., 2014; Wishart et al., 2007, 2018).

Statistical analysis was done on SPSS statistic for Windows, version 17.0 (SPSS Inc., Chicago, IL, USA). The Kolmogorov–Smirnov test and visual assessment were performed by quantile-quantile plotting that was used to determine data distribution. Results are presented as mean ± standard deviation for normally distributed data and median (range) for non-normally distributed data. Data comparison was done by Mann–Whitney *U*-test in both non-parametric data and independent *t*-test in parametric data. Serum metabolites were further analyzed by multivariate statistical analysis using MetaboAnalyst (http://www.metaboanalyst.ca) (Chong et al., 2018; Xia & Wishart, 2016). Partial least squares discriminant analysis (PLS-DA) was performed to identify variable metabolites. Results with *p*-value < 0.05 were considered statistically significant.

## Results

This current study on young adults consisted of a total of 46 subjects. The control group consisted of 23 subjects with BMI between 18.5 and 24.9 kg/m^2^. The OW group consisted of 23 subjects with BMI between 25.0 and 29.9 kg/m^2^. The characteristics of both groups are shown in Table 1. Dyslipidemia was found to be 52.17% in control group and 65.22% in the OW group.

**Table 1:** Characteristics and anthropometry of 46 subjects in control group and overweight group.

*N*	Control	Overweight	*p*-values
	23	23	
Gender (male/female)	7/16	13/10	–
Age (years)	22.17 ± 0.21	22.65 ± 0.45	<0.352
BMI (kg/m^2^)	21.45 ± 2.08	29.95 ± 1.96	<0.001^a^
Triglyceride (mg/dL)	77.91 ± 31.22	128.43 ± 55.83	0.036^a^
TC (mg/dL)	188.57 ± 47.11	207.17 ± 42.36	0.956^a^
HDL (mg/dL)	56.09 ± 15.15	47.65 ± 8.67	0.033^a^
LDL (mg/dL)	115.36 ± 36.89	134.76 ± 35.79	0.889^a^
Glucose (mg/dL)	82.00 (80.8–85.4)	91.00 (86.1–93.1)	0.004^b^
HbA1c (%)	5.10 (4.9–5.2)	5.40 (5.2–6.0)	0.002^b^

**Notes:**

a Independent student *t*-test.

b Mann–Whitney *U*-test. Data expressed as mean ± SD or median (range). BMI, body mass index; TC, total cholesterol; HDL, high density lipoprotein; LDL, low density lipoprotein; HbA1c, glycated hemoglobin.

**Figure 1:**
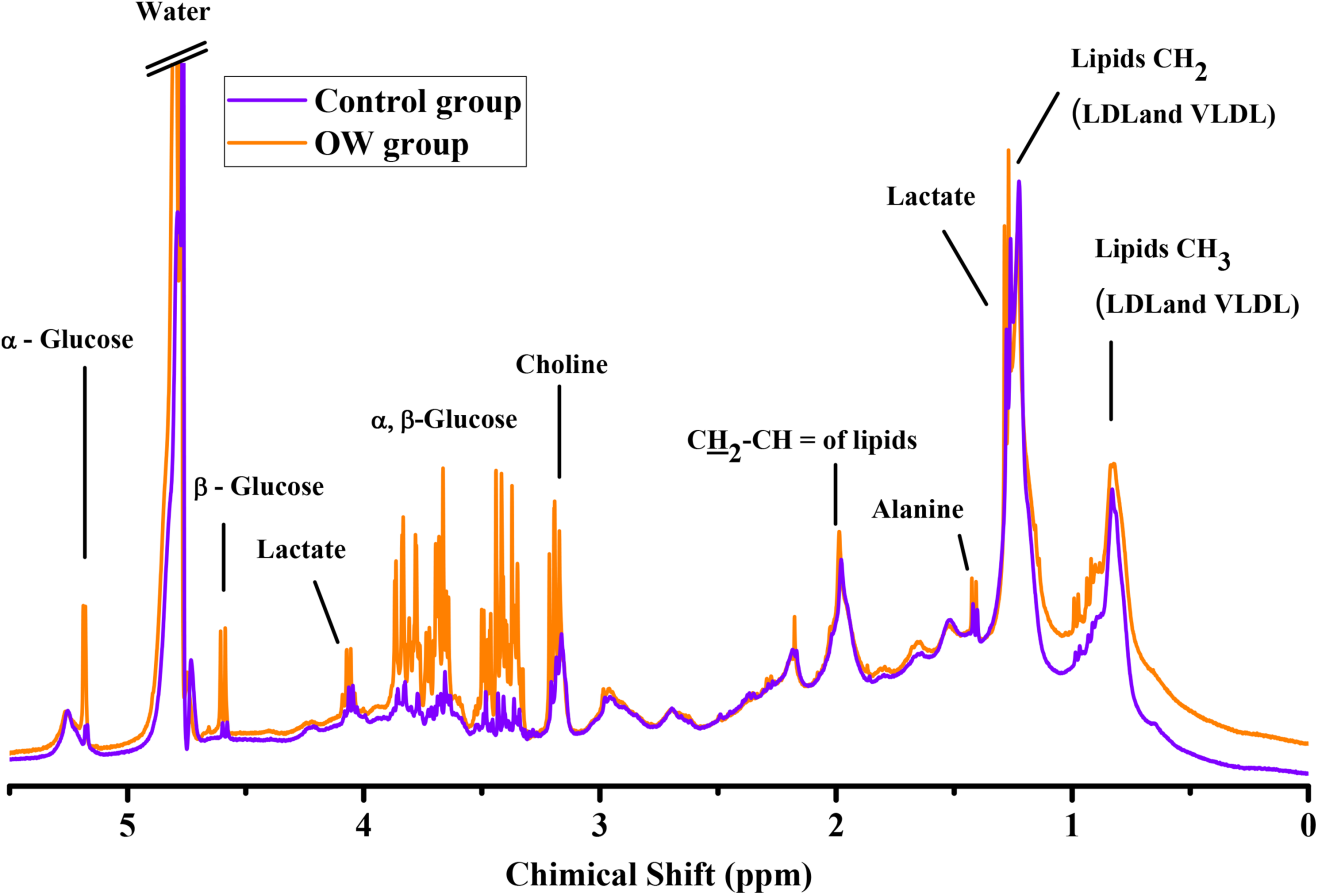
Overlay representative NMR serum metabolites spectra. The spectrum from the overweight group show slightly higher lipids, α-glucose and β-glucose, control group (purple line), and overweight group (OW, orange line).

A total of 46 NMR serum metabolites spectra were acquired from 23 subjects in the control group, and from 23 subjects in the OW group. Metabolite quantification was done by the integration of area under the peak of each metabolite of interest, and each metabolite was manually identified by its unique chemical shift. NMR spectrum showed nine distinguishing metabolites: CH_3_ lipids, CH_2_ lipids, lactate, alanine, CH_2_–CH= lipids, creatine, choline, α-glucose, and β-glucose. Chemical shift assignment of metabolite peaks is based on previously published research (Hu et al., 2001; Xia & Wishart, 2016). Lipids were calculated into total lipid levels by adding up the relative concentration of lipids at 0.9, 1.3, and 2.0 ppm. Additionally, α-glucose and β-glucose were calculated into total glucose. Representative NMR serum metabolites spectra of control group and OW group are shown in Fig. 1.

Lipids and glucose were calculated, and the results demonstrated that both total lipid and glucose were higher in the OW group. Statistical analysis indicates significantly different levels of CH_3_ lipids (*p* = 0.016), CH_2_ lipids (*p* < 0.0001), CH_2_–CH= lipids (*p* = 0.049), and total lipids (*p* < 0.0001). The metabolite quantification results of OW group showed higher α, β-glucose at 5.22, and 4.63 ppm with higher level lipids occurring at 0.9, 1.3, and 2.0 ppm. Other metabolites were altered when compared to control group, but did not do so in significantly different ways. However, creatine at 3.03 ppm was not found in all cases, therefore creatine was excluded from any further statistical analysis. The control group of metabolites were set to be a reference at 100% for normalization. Afterward, the percentage change in the OW group was then compared to control group and calculations were made (Table 2).

**Table 2:** The percentage change and trends of metabolite levels obtained in the overweight group when compared to the control group.

Metabolite	Chemical shift (ppm, δ)	Percentage change (%)	*p*-value
Lipids CH_3_	0.9	+26.49	0.016
Lipids CH_2_	1.3	+47.98	<0.0001
Lactate	1.33	+13.27	0.956
Alanine	1.48	−0.37	0.684
Lipids CH_2_–CH=	2.0	+16.15	0.049
Choline	3.19	−8.20	0.750
β-glucose	4.63	+6.92	0.974
α-glucose	5.22	+6.48	0.956
Total glucose	–	+6.74	0.974
Total lipid	–	+34.10	<0.0001

**Note:**

Significant levels obtained from Mann–Whitney *U*-test.

**Figure 2:**
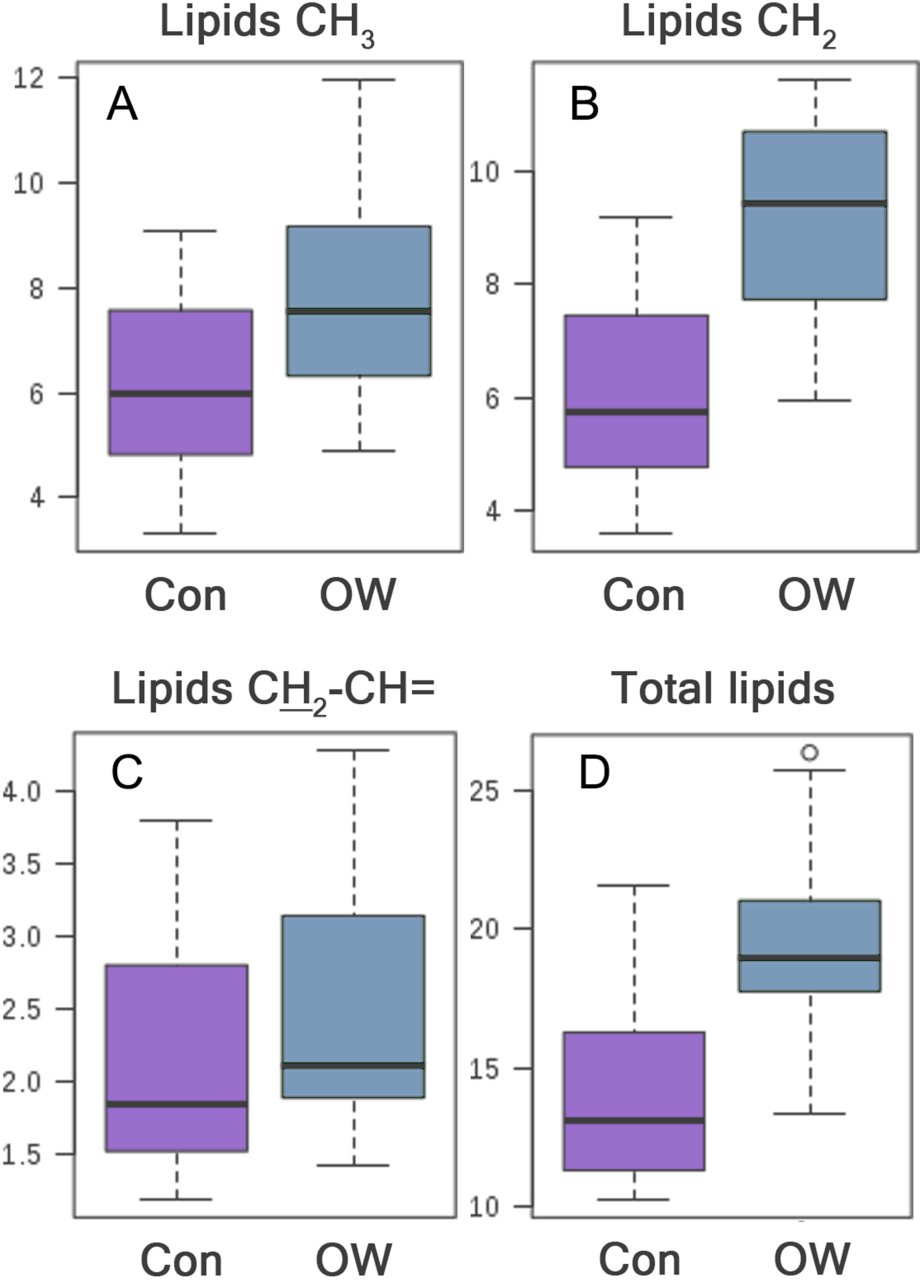
Box plot of metabolite intensity of lipids CH_3_, CH_2_, CH_2_CH= and total lipids between control group (Con) and overweight group (OW). The lipid intensity of serum lipids obtained from OW is significantly higher than control group, (A) with CH_3_ lipids (*p* = 0.016), (B) CH_2_ lipids (*p* < 0.0001), (C) CH_2_–CH= lipids (*p* = 0.049), (D) and total lipids (*p* < 0.0001).

The significantly higher CH_2_ lipids, CH_3_ lipids, and total lipids were consistent with biochemical analysis of blood that also showed significantly higher triglyceride in the OW group. Boxplots of significantly different metabolites are shown in Fig. 2. Afterward, statistically significant metabolites were then tested for correlation with blood biochemical test (Table 3).

**Table 3:** Pearson correlation between biochemical analysis with significantly different metabolites.

	Correlation with CH_3_		Correlation with CH_2_		Correlation with CH_2_–CH=	
	*r*	*p*-values	*r*	*p*-values	*r*	*p*-values
Glucose (mg/dL)	0.425	0.003	0.594	0.0001	0.328	0.026
HbA1c (%)	0.295	0.046	0.400	0.006	0.050	0.741
Triglyceride (mg/dL)	0.234	0.117	0.512	0.0001	0.131	0.387
TC (mg/dL)	0.195	0.195	0.277	0.063	0.093	0.539
LDL (mg/dL)	0.210	0.161	0.261	0.080	0.104	0.493
HDL (mg/dL)	−0.59	0.698	−0.088	0.562	−0.54	0.722

**Note:**

HbA1c, glycated hemoglobin; TC, total cholesterol; LDL, low density lipoprotein; HDL, high density lipoprotein; *r*, Pearson correlation coefficient.

**Figure 3:**
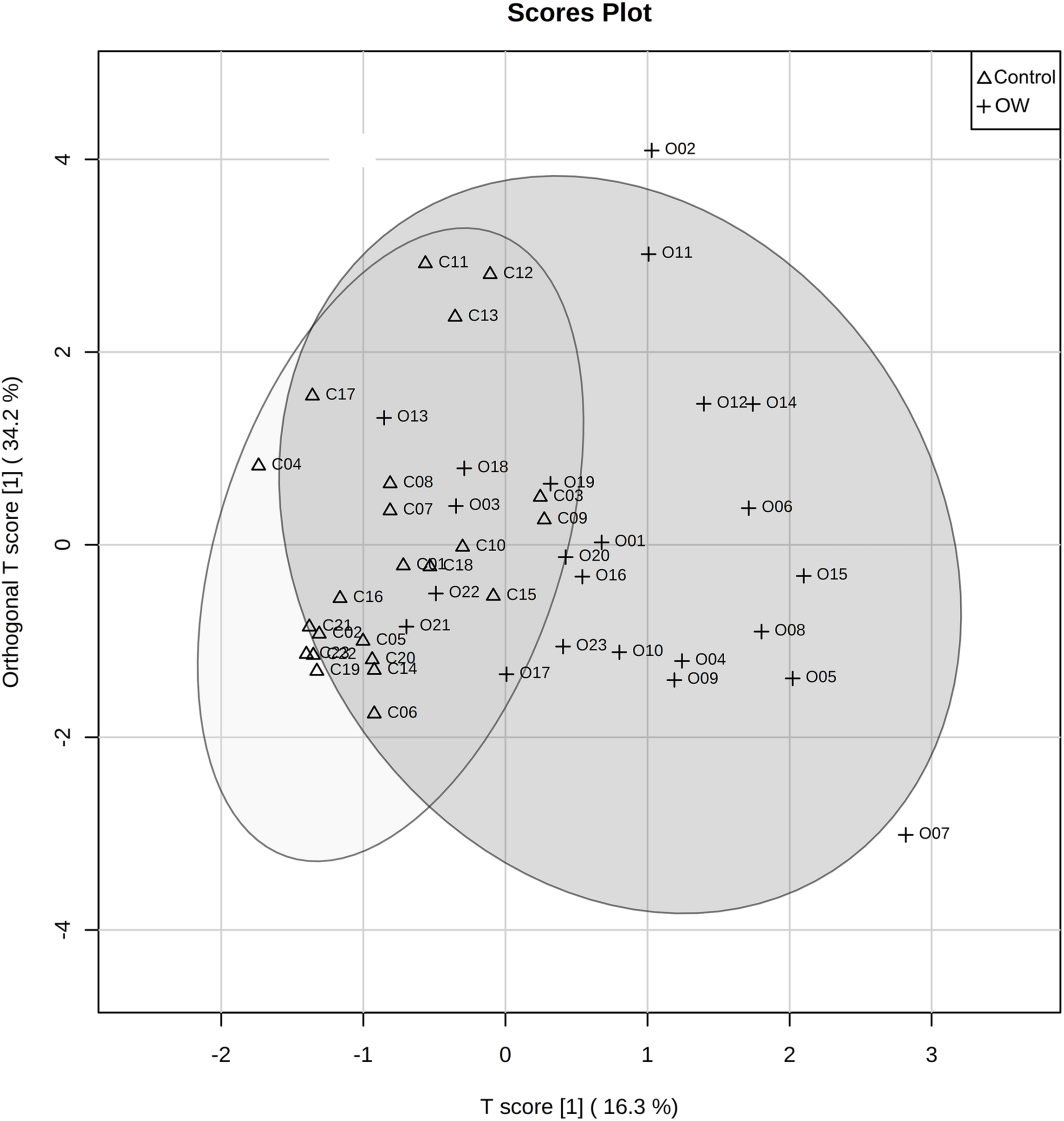
PLS-DA score plot comparing control group and overweight group. The figure reveals the discrimination of metabolites between two groups, control group (Con, triangle) and overweight group (OW, cross).

**Figure 4:**
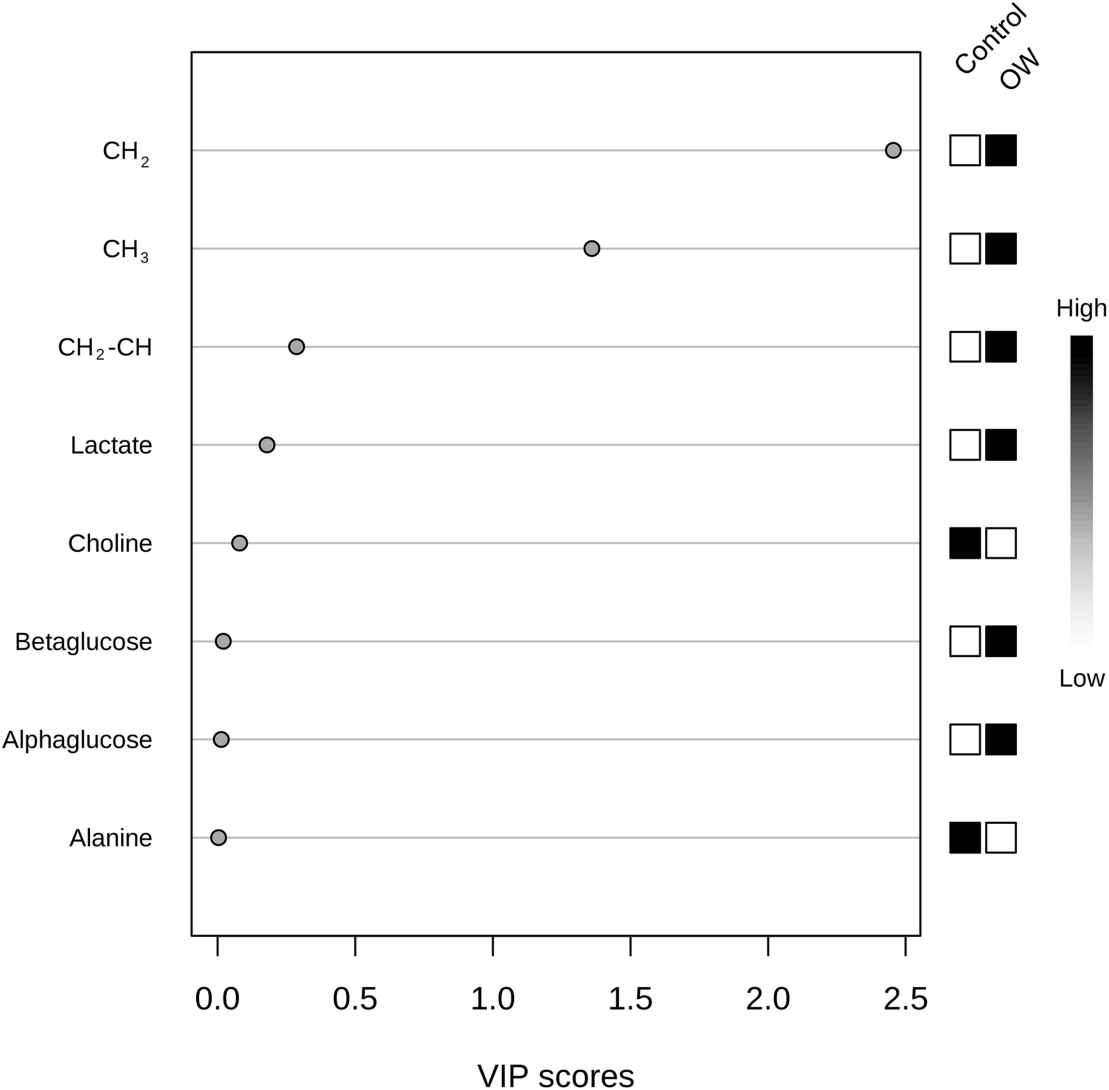
The variable importance in projection (VIP) scores plot from PLS-DA analysis of interested metabolites from control group (Con) and overweight group (OW). VIP score (higher than 1) shows that CH_2_ and CH_3_ are importance features metabolites. Black and white boxes on the right indicate the relative concentrations of each metabolite from each group.

Partial least squares discriminant analysis was used for analyzing serum metabolites between the control group and OW group. The PLS-DA scores plot reveals the distinctive separation between the two groups (Fig. 3). The measurement of variance importance of interested metabolites was done by Variable importance in projection (VIP) score to identify the potential biomarker that contributed to the model (Fig. 4).

The black and white colored boxes on the right side of VIP scores plot indicated a high or low relative concentration of metabolite in each group. The threshold for relevant metabolites selection is VIP equaling one or higher. Any metabolites with VIP score close to, or higher than one (1) can be considered to have significant effect on the given model. VIP scores, lipids CH_2_, and lipids CH_3_ were considered to be potential biomarkers that distinguished the OW group from the control group. These results were in agreement with the statistical test done by Man–Whitney *U*-test. However, Lipids CH_2_–CH= that was previously shown as statistically significant was not determined to be an important variable based on the PLS-DA analysis.

## Discussion

The prevalence of obesity and being OW among young adults is increasingly becoming evident. Despite having knowledge, extensive education, and degrees, young adults in this age group often exhibit unhealthy diets and behaviors (Gores, 2008; Peltzer et al., 2014). The unhealthy lifestyles found in this age group may result from physiological, physical, social, and environmental factors.

In this current study there was a high prevalence of dyslipidemia in both the control group and OW group. However, the triglyceride levels, HbA1c, and blood glucose levels were significantly higher in the OW group. This coincided with a significantly lower HDL in the OW group, as well. These results support the notion that high HbA1c levels in OW subjects have long-term effects from high glycemic levels and is a predictor for insulin resistance (Power & Thomas, 2011; Singh & Saxena, 2010). Alteration of HDL levels among OW and obese individuals is associated with obesity (Rashid & Genest, 2007; Sanossian et al., 2007). This result also agrees with previous studies of the association between HDL and insulin resistance in OW and obese individuals (Hirschler et al., 2015; McLaughlin et al., 2003).

The NMR results has found various alterations of serum metabolites taking place between the control group and OW group. Results show elevated lipids and glucose levels occurring in the OW group. In the OW group CH_3_ lipids, CH_2_ lipids, CH_2_–CH= lipids, and total lipids were statistically significantly higher when compared to control group. However, PLS-DA analysis determined that only CH_2_ lipids and CH_3_ lipids were potential biomarkers. The increased serum CH_2_ lipids and CH_3_ lipids which are aroused from LDL and VLDL is consistent with the biochemistry analysis obtained from venous blood that also demonstrates significantly elevated LDL content in the OW group. VLDL is the lipid that is responsible for balancing lipid levels in the liver and blood. Triglyceride accumulation in the liver and insulin resistance will activate VLDL overproduction. High VLDL released from the liver will result in higher LDLs, and from triglycerides and cholesterol ester exchanges taking place between VLDL and LDL via the cholesterol ester transfer protein (CETP)-mediated pathways (Trajkovska & Topuzovska, 2017). The CETP-mediated pathway has found to play a crucial role in dyslipidemia among the obese and insulin resistant individuals. VLDL is one of the important features of metabolic impairment, and is associated with insulin resistance in the liver, the muscles, and in adipose tissue. Some studies suggest that the CETP-mediated pathway is associated with a higher risk of cardiovascular disease (Sanossian et al., 2007; Van Gaal, Mertens & De Block, 2006). Moreover, VLDL is also associated with LDL receptor impairment, which results in a low VLDL clearance rate (Howard et al., 2000).

There is a significant relationship of glucose with all of lipids evident from the NMR study. CH_2_ and CH_3_ lipids show correlation with glucose, however CH_2_–CH= lipids do not. This result agrees with VIP score assessment of metabolites from NMR studies. The CH_2_ lipids also show moderate correlation (*r* = 0.512) with blood triglycerides, which can be explained by the fact that CH_2_ is also included in the lipid signal arising from triglycerides in the blood.

The alteration of other metabolites was not significantly different, but is nonetheless noteworthy. These changes in metabolite level may reveal the mechanisms underlying pathogenesis. Choline is an essential nutrient for maintaining cells and the mitochondrial membrane (Vance, 2008). Various studies have found that choline is associated with obesity. Furthermore, choline also can be phosphorylated to phosphatidylcholine which is an important metabolite for transporting LDL, and serves as an intermediary to maintain a balance between fat in the liver and plasma. The alteration of choline in OW group may suggest a modification of lipid metabolism taking place (Vance, 2008; Yao & Vance, 1988). However, Alanine levels remained the same in both groups.

Higher total glucose (α and β glucose) in the OW group is also remarked. This shows an increased risk of OW whenever blood glycemic levels increase. Elevated lactate was also found in this study, which are metabolites that are associated with the gluconeogenesis process. Metabolomic studies on obese subjects have found higher levels of lactate in the blood. This may indicate an increased anaerobic metabolism and glucose production as lactate is a precursor of gluconeogenesis (Newgard et al., 2009). The combination of evaluated levels of both glucose and lactate in serum may be a sign of pyruvate-dehydrogenase pathway and liver function that is associated with blood lipid impairment in the obese. In accordance with present results, previous research suggested similar connections of changes taking place in the pyruvate-dehydrogenase pathways (Amathieu et al., 2011).

This current study has both limitations and strengths. One limitation is that the health and diet behavior are self-reported. This study was done in a particular area, despite the effort to randomly from a wider area. Moreover, young adults in this age group often decline to participate due to conflicts in schedules. This difficultly in recruiting subjects in this age group is widely acknowledged in various studies (Hu et al., 2001; Huang et al., 2003; Peltzer et al., 2014; Vadeboncoeur, Townsend & Foster, 2015). Despite these limitations, the strength of this study is that it includes analysis on how OW effects metabolites of emerging adults by ^1^H NMR technique. This is addressing a significant gap in understanding about OW among the young adults concerning the systemic alteration taking place in this age group. Although this current study is based on a small sample of subjects, the findings have drawn together various interesting subjects on the effects of BMI and how it contributed to metabolite and health status. Furthermore, it shows how OW is a high-risk factor of metabolic syndrome in young adults. Future studies on this topic are therefore recommended as this age group is at a high risk for developing metabolic diseases. Future research might explore similar questions in longitudinal studies with larger groups of subjects, while addressing the ethnic, family medical history, and the various risks factors on metabolic effect.

## Conclusions

This study suggests that OW contributes to a systemic modification of metabolism and may lead to higher risks of metabolic diseases in young adults. ^1^H NMR metabolite analysis shows the alteration of metabolic levels and increased levels of lipids, which are used as unique biomarkers to identify OW subjects from the normal weight groups. These findings also suggest that young adults should be encouraged to maintain healthy weight and behaviors, as they are a key age group to focus on for better metabolic disease prevention.

## Supplemental Information

10.7717/peerj.7137/supp-1 Supplemental Information 1 NMR serum metabolites were acquired from the control group and from the overweight group. NMR metabolites: CH_3_ lipids, CH_2_ lipids, lactate, alanine, CH_2_–CH= lipids, creatine, choline, α-glucose, and β-glucose.

10.7717/peerj.7137/supp-2 Supplemental Information 2 Waist to Hip ratio and HDL of 46 subjects in the control group and the overweight group reported. Data expressed as mean ± SD. *P*-value obtained from independent student *t*-test; HDL: high density lipoprotein; W/H ratio: waist to hip ratio.

## Additional Information and Declarations

### Data Availability

The following information was supplied regarding data availability: The raw data is available as Supplemental Files.
