# Supplementary material for: Effects of obesity on the lipid and metabolite profiles of young adults by serum 1H-NMR spectroscopy
Source: PeerJ. 2019 Jun 20;7:e7137. doi: 10.7717/peerj.7137 (PMC6589329; doi:10.7717/peerj.7137)
Supplement: Supplemental Information 2 — Data expressed as mean ± SD. P-value obtained from independent student t-test; HDL: high density lipoprotein; W/H ratio: waist to hip ratio. [file peerj-07-7137-s002.docx]

|  | Female | | *P-value* | Male | | *P-value* |
| --- | --- | --- | --- | --- | --- | --- |
|  | Control | Overweight |  | Control | Overweight |  |
| N | 16 | 10 |  | 7 | 13 |  |
| HDL | 57.35±16.27 | 50.00±9.38 | 0.278 | 52.50±11.91 | 46.63±8.45 | 0.208 |
| W/H ratio | 0.83±0.05 | 0.90±0.05 | 0.005 | 0.82±0.07 | 0.94±0.06 | 0.001 |

TableS1. Waist to Hip ratio and HDL of 46 subjects in control group and overweight group reported

Data expressed as mean±SD.

P-value obtained from independent student t- test; HDL: high density lipoprotein; W/H ratio: waist to hip ratio.
